# Supplementary material for: Detection of Anaplasma phagocytophilum DNA in Deer Keds: Massachusetts, USA
Source: Insects. 2025 Jan 4;16(1):42. doi: 10.3390/insects16010042 (PMC11765709; doi:10.3390/insects16010042)
Supplement: Supplementary file 1 [file insects-16-00042-s001.zip › insects-3387293-supplementary.pdf]

Table S1. Primers and probes used to detect pathogens.

Target	Type	Sequence (5’-3’)
Borrelia genus	Forward primer	GCTTCGCTTGTAGATGAGTCTGC
	Reverse primer	CCGTAGGAGTCTGGACCGTATC
	Probe	TCCAGTGTGACCGTTCACCCTCTCAGGC
Borrelia burgdorferi Sensu Lato	Forward primer	ATAGGTCTAATATTAGCCTTAATAGCAT
	Reverse primer	AGATCGTACTTGCCGTCTT
	Probe	aagc+Aaa+Atgtt+Agc+Agccttga
Borrelia miyamotoi	Forward primer	AGACATAGTTCTAACAAAGGACAATATTCC
	Reverse primer	AGTTCAGTTAGTGTGAAGTCAGTAGC
	Probe	TGCACGACCCAGAAATTGACACAACCACAA
Borrelia mayonii	Forward primer	TTGGTTGCATCCATAGATGAACTTG
	Reverse primer	CGGTTGATATTGTATAGATTCCTGATAGC
	Probe	CCATTCTTACCTGCTTCATTCCCCAACCCATTATTTT
Babesia microti	Forward primer	GATTTGGAACCTGGCACCATG
	Reverse primer	AATGACCCTTAGCCCAATTATTTCC
	Probe	ATCTGGCCCATACGGTGAATTGTTTCGC
Anaplasma	Forward primer	ATGGAAGGTAGTGTTGGTTATGGTATT
	Reverse primer	TTGGTCTTGAAGCGCTCGTA
	Probe	TGGTGCCAGGGTTGAGCTTGAGATTG
Ehrlichia muris	Forward primer	TACCTAATTCTTCTCAAGAGATTCAGTTG
	Reverse primer	ATGATGATACTGCGAACAACTATAAGAG
	Probe	ATATTGATAAAAGAGTCAGTGTTGATCCGTATGAGTTAGGGTT
