# Supplementary figures and images for: Detection of Anaplasma phagocytophilum DNA in Deer Keds: Massachusetts, USA
Source: Insects. 2025 Jan 4;16(1):42. doi: 10.3390/insects16010042 (PMC11765709; doi:10.3390/insects16010042)

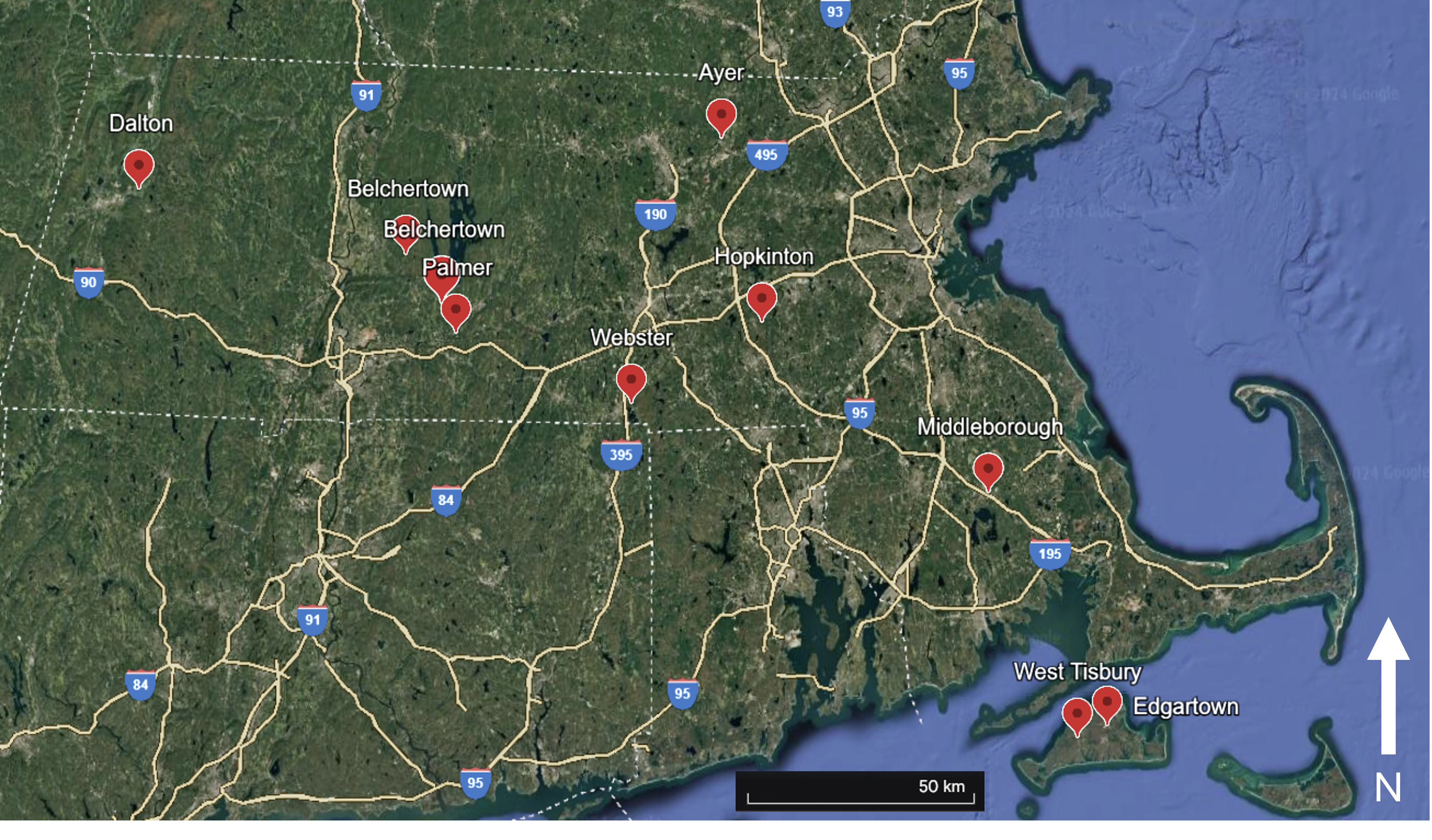

Supplement: Supplementary file 1 [file insects-16-00042-s001.zip › insects-3387293-supplementary.png]
